# Metabolic Health Is More Closely Associated with Coronary Artery Calcification than Obesity

**DOI:** 10.1371/journal.pone.0074564

**Published:** 2013-09-11

**Authors:** Eun-Jung Rhee, Mi Hae Seo, Jong Dae Kim, Won Seon Jeon, Se Eun Park, Cheol-Young Park, Ki-Won Oh, Sung-Woo Park, Won-Young Lee

**Affiliations:** 1 Department of Endocrinology and Metabolism, Kangbuk Samsung Hospital, Sungkyunkwan University School of Medicine, Seoul, Republic of Korea; 2 Department of Endocrinology and Metabolism, Soonchunhyang University Gumi Hospital, Gumi, Republic of Korea; University of Groningen, Netherlands

## Abstract

**Background:**

Recent studies have suggested that metabolic health may contribute more to the atherosclerosis than obesity. The aim of this study is to compare coronary artery calcium scores (CACS) among patients with different metabolic health and obesity status.

**Methods:**

A health-screening program of 24,063 participants (mean age 41 years) was conducted, and CACS was assessed by multi-detector computerized tomography (MDCT). Being metabolically healthy was defined as having fewer than two of the following risk factors: high blood pressure, high fasting blood glucose, high triglyceride, low high-density lipoprotein cholesterol, highest decile of homeostasis model assessment-insulin resistance (HOMA-IR) index, and highest decile of high-sensitivity C-reactive protein (hs-CRP). Obesity status was defined as body mass index (BMI) higher than 25 kg/m^2^. Analyses were performed in four groups divided according to metabolic health and obesity: metabolically healthy non-obese (MHNO), metabolically healthy obese (MHO), metabolically unhealthy non-obese (MUHNO), and metabolically unhealthy obese (MUHO).

**Results:**

Mean values of CACS in the four groups were significantly different, except those between MHNO and MHO and between MUHNO and MUHO. When multinomial logistic regression analysis was performed with five CACS categories as the dependent variables and after adjusting for age, sex, and smoking status, the MHO, MUHNO, and MUHO groups showed significantly increased odds ratio for increasing CACS categories compared with no calcification status (5.221 for CACS >400 in MUHO group with 95% CI 2.856∼5.032 with MHNO group as the reference). When other variables including the metabolic parameters were included in the same model, the risks were attenuated.

**Conclusion:**

Metabolic health is more closely associated with subclinical atherosclerosis than obesity as assessed by CACS.

## Introduction

Obesity is the main cause of various metabolic diseases and its ultimate consequence is increased risk of cardiovascular disease [1]. Being obese not only means excess body fat content but also having the consequences of “inflamed fat,” or metabolic derangements such as diabetes, insulin resistance, vascular inflammation, and atherosclerosis [2].

Recent studies have suggested that an obesity phenotype may present without these metabolic derangements; thus, obesity is not necessarily equivalent to poor metabolic health [3]. This new concept led to the creation of the new term, “metabolically healthy obesity (MHO),” which refers to obese subjects who satisfy the current definition of obesity without satisfying the criteria for being metabolically unhealthy, such as having insulin resistance, elevated blood pressure, dyslipidemia, or elevated surrogate markers of systemic inflammation [4]. Although no unified definition of being “metabolically unhealthy” has been established, proposed definitions include adopting the components of the metabolic syndrome with the addition of an insulin resistance index or serum inflammatory markers. Unfortunately, there are not many studies that examine the clinical significance of metabolically healthy obesity.

Screening for subclinical atherosclerosis is an important issue for the early detection and prevention of overt cardiovascular disease (CVD). Recently, there have been many studies of non-invasive measures for the detection of subclinical atherosclerosis [5]. Among them, coronary artery calcium score (CACS) determined by computed tomography (CT) is an excellent tool for measuring the plaque burden in subjects at high risk for CVD [6]–[8]. A direct correlation was reported between CACS severity and the risk of future cardiovascular events, assessed by the Framingham Risk Score [9].

The participants provided their written informed consent for the use of their health screening data for this research. The design, protocol, and consent procedure of this study were reviewed and approved by the Institutional Review Board (IRB) of Kangbuk Samsung Hospital and are in accordance with the Helsinki Declaration of 1975. After the review and acceptance of the study protocol by IRB, specific dataset for this study were released by the data management group in KSHS after deleting the personal information of the participants.

There are few studies that have analyzed the impact of metabolically healthy obesity on cardiovascular risk. A recent prospective study with median 14 years of follow-up reported that metabolically healthy obese subjects had lower risk of all-cause mortality and non-fatal and fatal CVD than their metabolically unhealthy obese peers [10]. Another study showed that metabolically unhealthy subjects were at elevated risk for CVD compared with their metabolically healthy counterparts regardless of their obesity status after an average of 7 years of follow-up [11]. In addition, when the subclinical CVD burden was compared between metabolically “benign” and “at-risk” obese women, the frequency of women with increased CAC and aortic calcification was higher in the metabolically “at-risk” group compared with the “benign” obese group [12]. This study was limited by its small study population and lack of male study subjects.

We aimed to compare the CACS between four groups, divided by obesity status and metabolic health, including insulin resistance index, in a large, health-screening cohort. In addition, we analyzed the coronary calcification in these groups to clarify whether metabolic health or obesity is associated with coronary artery calcification.

## Methods

### Subjects

This cross-sectional study was a part of the Kangbuk Samsung Health Study (KSHS), in which subjects participated in a medical health checkup program at the Health Promotion Center of Kangbuk Samsung Hospital, Sungkyunkwan University, Seoul, Korea. The purpose of the medical health checkup program is to promote the health of employees through regular health checkups and to enhance early detection of existing diseases. Most of the examinees are employees and family members of various industrial companies from around the country. The costs of the medical examinations are largely paid by employers, and a considerable proportion of the examinees undergo examinations annually or biannually.

**Figure 1:**
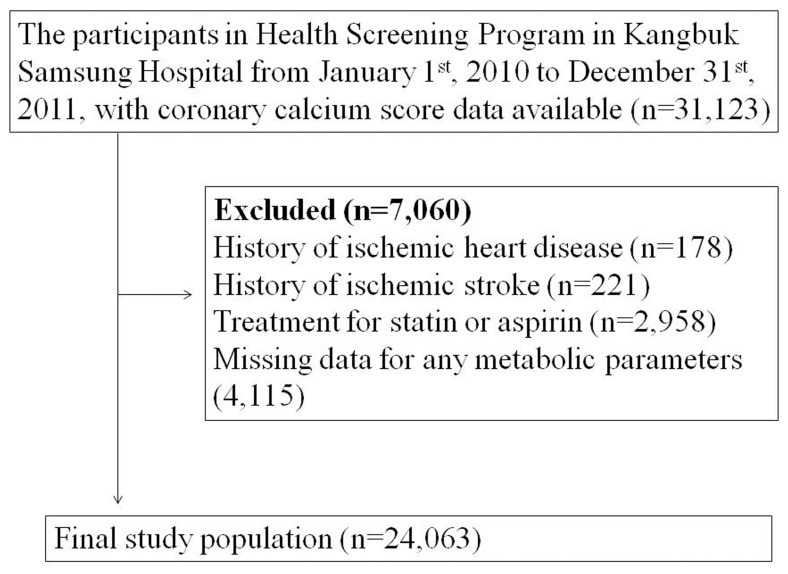
Selection of the study population.

Of the 31,123 subjects who participated in the medical checkup program from January 2010 to December 2011, various exclusion criteria were applied. We excluded subjects with a self-reported history of ischemic heart disease (*n* = 178) or ischemic stroke (*n* = 221), subjects who were taking a statin or aspirin (*n* = 2,958), and subjects with any missing data (*n* = 4,115). These rigorous exclusions resulted in a final study population of 24,063 subjects (Figure 1).

### Anthropometric and Laboratory Measurements

Height and weight were measured twice and then averaged. The body mass index (BMI) was calculated by dividing the weight (kg) by the square of the height (m). Blood pressure was measured using a standardized sphygmomanometer after five minutes of rest. The waist circumference (WC) was measured in the standing position, at the midpoint between the anterior iliac crest and lower border of the last palpable rib by a single examiner.

All of the subjects were examined after an overnight fast. Serum calcium level was measured by the O-cresolphethalein complexone method (Hitachi Modular D2400; Roche, Tokyo, Japan). The hexokinase method was used to test fasting glucose concentrations (Hitachi Modular D2400; Roche, Tokyo, Japan). Fasting insulin concentrations were determined by electrochemiluminescence immunoassay (Hitachi Modular E170; Roche, Tokyo, Japan). An enzymatic calorimetric test was used to measure the total cholesterol and triglyceride concentrations. The selective inhibition method was used to measure the level of high-density lipoprotein cholesterol (HDL-C), and a homogeneous enzymatic calorimetric test was used to measure the level of low-density lipoprotein cholesterol (LDL-C). Serum high-sensitivity C-reactive protein (hs-CRP) levels were measured using a nephelometric assay with a BNII nephelometer (Dade Behring, Deerfield, IL).

The presence of diabetes mellitus was determined by answers to the participant self-questionnaire and the diagnostic criteria of the American Diabetes Association [13]. The presence of hypertension was defined as blood pressure (BP) ≥140/90 mm Hg or presently taking anti-hypertensive medication, according to the criteria recommended by the seventh report of the Joint National Committee on prevention, detection, evaluation, and treatment of high BP (JNC 7) [14]. Smoking status was determined by the questionnaire. A smoker was defined as a subject who had ever smoked at least five packs of cigarettes in his life. Doing exercise was defined as regular exercise of moderate intensity every week.

Insulin resistance was measured using the homeostatic model of the assessment of insulin resistance (HOMA-IR) and was obtained by applying the following formula: HOMA-IR = fasting insulin (IU/mL) × fasting blood glucose (mmol/L)/22.5 [15].

### Measurement of Coronary Artery Calcium Score

Multi-detector computed tomography (MDCT) for coronary calcium scoring was undertaken by a 64-slice, spiral computed tomography scan (GE Health Care, Tokyo, Japan). The severity of coronary artery calcification was assessed by Agatston score [16]: 0, 1–10, 11–100,101–400 or >400. A total CACS was determined by the sum of the individual scores for the four major epicardial coronary arteries.

### Definition of Being Metabolically Healthy Obese

Obesity phenotypes were defined based on BMI category (non-obese <25 kg/m^2^, obese ≥25 kg/m^2^). In 2000, the World Health Organization Western Pacific Region suggested revised Asia–Pacific criteria of obesity in Asian populations using reduced values for body mass index (BMI ≥25 kg/m^2^ in both sexes) [17]. In addition, obesity was also defined by WC category; men ≥90 cm, women ≥85 cm for Koreans [18].

Being metabolically healthy was defined as having less than two of the following risk factors [19]:

1) Systolic blood pressure ≥130 mmHg and/or diastolic blood pressure ≥85 mmHg, or on antihypertensive treatment.

2) Triglyceride ≥150 mg/dl.

3) Fasting glucose ≥100 mg/dl or being treated for diabetes.

4) HDL-cholesterol <40 mg/dL in men, <50 mg/dL in women.

5) HOMA-IR ≥90^th^ percentile (≥2.6).

6) Hs-CRP≥90^th^ percentile (≥0.22 mg/L).

According to these criteria, participants were divided into 4 groups:

1) Metabolically healthy, non-obese (MHNO): BMI <25 kg/m^2^and <2 metabolic risk factors.

2) Metabolically healthy, obese (MHO): BMI ≥25 kg/m^2^ and <2 metabolic risk factors.

3) Metabolically unhealthy, non-obese (MUHNO): BMI <25 kg/m^2^ and ≥2 metabolic risk factors.

4) Metabolically unhealthy, obese (MUHO): BMI ≥25 kg/m^2^ and ≥2 metabolic risk factors.

### Statistical Methods

All data are presented as mean ± standard deviation and were analyzed using SPSS Windows version 18.0 (SPSS Inc., Chicago, IL, USA). Bivariate correlation analyses were performed between CACS+1 and the variables of interest to use the information contained in CACS more fully. Pearson’s correlation analysis and partial correlation analyses were performed to adjust for age and sex. Comparison of the parameters among the four groups divided by metabolic health and obesity status was analyzed by one-way ANOVA test, and analysis of covariance (ANCOVA) test was performed to adjust for age and sex. Comparisons of the prevalence of subjects with different CACS categories, of proportion of subjects with CACS >0 among the four groups, and of the prevalence of metabolically unhealthy subjects among the groups, divided by CACS categories were performed with the chi-square test. Nonparametric comparisons of the medians for the variables between the groups were performed with Kruskal-Wallis H test and Mann-Whitney U test for post-hoc analyses with Bonferroni correction. Multinomial logistic regression analyses with the CACS categories as the dependent variable were performed with other confounding variables included in the model. Significance was defined as *p*<0.05.

## Results

### Study Population

**Figure 2:**
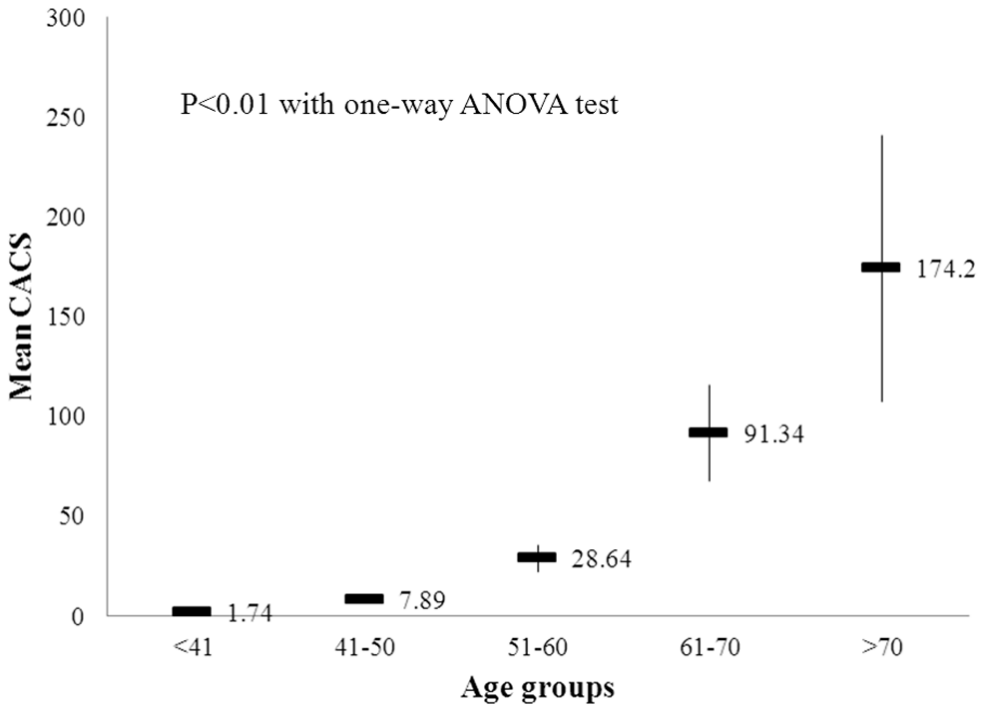
Coronary artery calcium scores in different age groups.

General characteristics of the participants are presented in Table 1. Mean age of the participants was 41 years (range 23–89 years). Mean value of BMI was 24.4 kg/m^2^, and 82% of the participants were male. Of the study population, 5.5% were being treated for diabetes or satisfied the diagnostic criteria for diabetes, 27.8% were hypertensive, and 51.9% had ever smoked more than 5 total packs of cigarettes in his or her life. Only 12.6% of the study population had significant coronary artery calcification (CACS >0). Mean CACS significantly increased with age (Figure 2). Among the five categories of CACS severity, 85.7% of men and 94.9% of women had CACS of 0 (Figure S1).

**Table 1:** General characteristics of the participants.

N = 24,063	Mean±SD (median, interquartile range)	
Age (years)	41.3±7.0	
Sex: Male (%)	4340 (18)	
Body weight (kg)	71.2±11.8	
Body mass index (kg/m^2^)	24.4±3.1	
Waist circumference (cm)	85.1±8.3	
Systolic blood pressure (mmHg)	117±13	
Diastolic blood pressure (mmHg)	75±9	
Calcium (mg/dL)	9.4±0.3	
Fasting blood glucose (mg/dL)	97.4±16.1	
Total cholesterol (mg/dL)	202.0 (179, 226)	
Triglyceride (mg/dL)	116 (81, 169)	
HDL-C (mg/dL)	51 (44, 61)	
LDL-C (mg/dL)	126 (105, 148)	
Hs-CRP (mg/L)	0.12±0.3	
Fasting insulin (uIU/mL)	5.9±3.7	
HOMA-IR	1.45±1.1	
Proportion of subjects with coronary artery calcification (%)*	3038 (12.6)	
Proportion of subjects with diabetes (%)	1383 (5.7)	
Proportion of subjects with hypertension (%)	6689 (27.8)	
Proportion of subjects who has ever smoked (%)	12495 (51.9)	
Proportion of subjects who do regular exercise with moderate intensity (%)	4276 (7.8)	

SD, standard deviation; HDL-C, high-density lipoprotein cholesterol; LDL-C, low-density lipoprotein cholesterol; hs-CRP, high-sensitivity C-reactive protein; HOMA-IR, homeostasis model assessment index-insulin resistance.

* Presence of coronary artery calcification was defined by CACS >0.

### Correlation of CACS with Variable Parameters

To use the information contained in CACS more fully, bivariate correlation analyses were performed between CACS+1 and multiple variables. Age showed the highest correlation with CACS+1 (Table 2). Most of the parameters showed positive correlations with CACS except HDL-C. When partial correlation analyses were performed with adjustment for age and sex, most of the parameters showed significant correlations with CACS+1 except serum calcium level.

**Table 2:** Correlation analyses of CACS+1 with various parameters.

	Correlation coefficient	Age- and sex-adjusted
Age	0.191*	–
Body weight	0.019*	0.023†
Body mass index	0.032*	0.020†
Waist circumference	0.044†	0.023†
Systolic blood pressure	0.056*	0.038†
Diastolic blood pressure	0.046*	0.022*
Calcium	−0.003	0.016*
Glucose	0.088*	0.059†
Total cholesterol	0.021*	0.002
Triglyceride	0.033*	0.014*
HDL-C	−0.026†	−0.010
LDL-C	0.021*	0.001
Hs-CRP	0.006	0.003
Insulin	0.019*	0.028†
HOMA-IR	0.040*	0.040†

CACS, coronary artery calcium score; HDL-C, high-density lipoprotein cholesterol; LDL-C, low-density lipoprotein cholesterol; hs-CRP, high-sensitivity C-reactive protein; HOMA-IR, homeostasis model assessment index-insulin resistance.

* p<0.01,

† p<0.05.

### The Relationship of Metabolic Health Status and Coronary Calcification

Subjects were divided into four groups according to metabolic health and obesity status as follows (Table 3): 10,838 (45.0%) subjects in MHNO group, 3,389 (18.2%) subjects in MHO group, 3,471 (14.4%) subjects in MUHNO group, and 5,365 (22.3%) subjects in MUHO group.

**Table 3:** Comparison of variables between the groups divided according to metabolic health and obesity status.

Variables	MHNO (%)	MHO (%)	MUHNO (%)	MUHO (%)	*P* value*
(N = 24,063)	10,838 (45.0)	4,389 (18.2)	3,471 (14.4)	5,365 (22.3)	
Age (years)	40.7±7.0^†^	40.7±6.8^†^	43.3±7.7	41.9±6.6	<0.01
Sex: Male (%)	7852 (72.4)	3959 (90.2)	2946 (84.9)	4965 (92.5)	<0.01
Body weight (kg)	63.8±8.6	78.8±8.1	67.3±7.4	82.2±9.7	<0.01
Body mass index (kg/m^2^)	22.2±1.8	26.8±1.7	23.1±1.4	27.8±2.4	<0.01
Waist circumference (cm)	63.8±8.6	78.8±8.1	67.3±7.4	82.2±9.7	<0.01
Systolic blood pressure (mmHg)	111.8±11.1	116.1±10.4	120.6±12.9	123.6±12.3	<0.01
Diastolic blood pressure (mmHg)	71.2±8.2	74.4±8.0	77.4±9.6	79.7±9.3	<0.01
Calcium (mg/dL)	9.37±0.33	9.39±0.31	9.45±0.33^†^	9.46±0.31^†^	<0.01
Fasting blood glucose (mg/dL)	92.4±9.3	93.6±8.3	105.1±22.2^†^	105.6±21.0^†^	<0.01
Total cholesterol (mg/dL)	197.4±33.8	206.7±33.9^†^	206.8±37.0^†^	213.5±37.4	<0.01
Triglyceride (mg/dL)	97.7±45.1	121.4±55.9	184.2±120.0	209.9±121.0	<0.01
HDL-C (mg/dL)	59.0±10.5	52.6±10.5	48.7±12.2	45.4±10.0	<0.01
LDL-C (mg/dL)	121.2±31.0	133.5±30.5	128.7±32.7	135.4±33.2	<0.01
Hs-CRP (mg/L)	0.08±0.25	0.11±0.3	0.18±0.50^†^	0.18±0.42^†^	<0.01
Fasting insulin (uIU/mL)	4.18±2.05	5.79±2.59	6.19±3.08	9.17±4.89	<0.01
HOMA-IR	0.96±0.49	1.34±0.61	1.61±0.92	2.42±1.52	<0.01
CACS	3.94±35.6^†^	6.49±51.9^†^	13.14±73.58^‡^	14.00±84.10^‡^	<0.01
Proportion of subjects with diabetes (%)	167 (1.5)	74 (1.7)	399 (11.5)	742 (13.8)	<0.01
Proportion of subjects with hypertension (%)	1150 (10.6)	694 (15.8)	1760 (50.7)	3085 (57.5)	<0.01
Proportion of subjects who has ever smoked (%)	4721 (43.6)	2464 (56.1)	1941 (55.9)	3369 (62.8)	<0.01
Proportion of subjects who do regular exercisewith moderate intensity (%)	2004 (18.5)	860(19.6)	562 (16.2)	850 (15.8)	<0.01

MHNO, metabolically healthy non-obese; MHO, metabolically healthy obese; MUHNO, metabolically unhealthy non-obese; MUHO, metabolically unhealthy obese; high-density lipoprotein cholesterol; LDL-C, low-density lipoprotein cholesterol; hs-CRP, high-sensitivity C-reactive protein; homeostasis model assessment index; CACS, coronary artery calcium score.

* *P* values for one-way ANOVA among the four groups.

†,‡ No differences between the groups with same footnotes in post-hoc analyses.

When parameters were compared among the groups, metabolically unhealthy groups were older and had worse metabolic parameters (Table 3). Metabolically unhealthy groups were generally more obese with higher mean BMI and WC compared to metabolically healthy peer groups. For the lipid profiles, mean LDL-C level, which is not used for categorization of metabolic health, was higher in obese groups than in non-obese groups, regardless of metabolic health. Mean total cholesterol level, another atherosclerotic risk factor that is not included in the categorization of metabolic health, was not significantly different in post-hoc analysis between MHO and MUHNO groups, suggesting a more important role of obesity than that of metabolic health on total cholesterol levels. These results were consistently significant even after adjustment for age and sex with ANCOVA test.

Metabolically unhealthy groups had higher proportions of subjects with diabetes and hypertension. Metabolically unhealthy subjects tended to exercise less, demonstrated by a smaller portion of subjects in their groups answering that they exercised regularly with moderate intensity compared with metabolically healthy counterparts (Table 3). More metabolically unhealthy subjects have smoked or were currently smoking compared with metabolically healthy subjects (Table 3).

**Figure 3:**
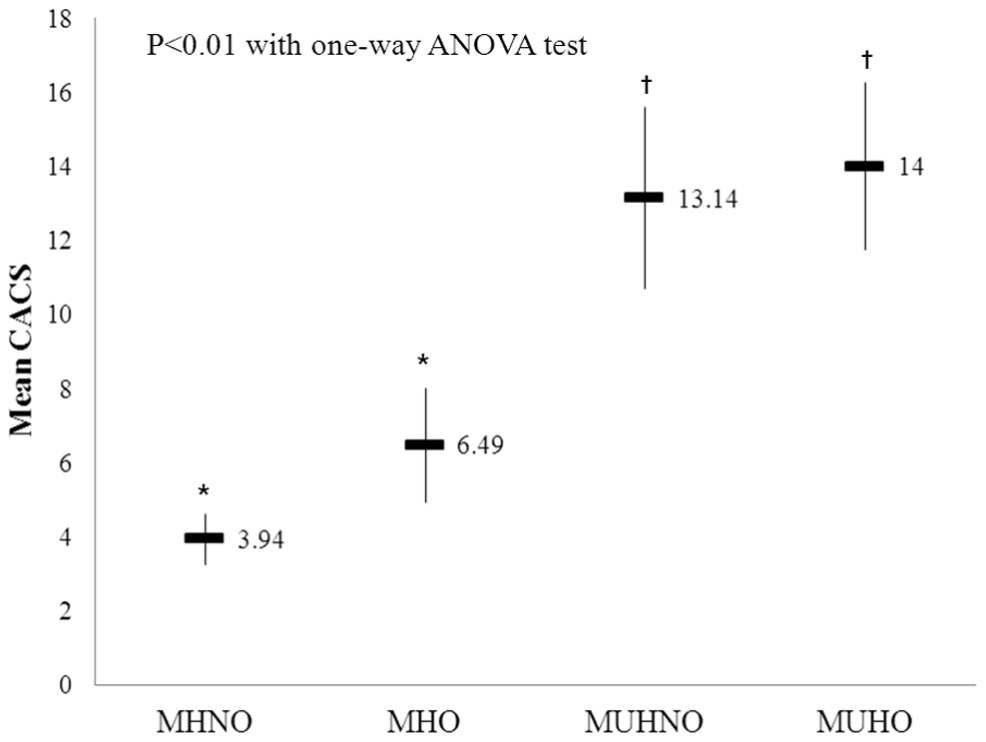
Comparison of coronary artery calcium score according to the four groups divided by metabolic health and obesity status. ^*,†^Same footnotes denote no differences between the designated groups.

The proportion of subjects with significant coronary artery calcification (CACS >0) was the highest in MUHO group and the lowest in MHNO group (20.3 vs. 7.9%) (Table S1). When the mean value of CACS was compared among the four groups, mean CACS was the highest in MUHO group and the lowest in MHNO group (Table 3, Figure 3). In post-hoc analysis, there were no differences between the groups with the same metabolic health status. That is, MHNO and MHO as well as MUHNO and MUHO were not statistically different, suggesting that within the same metabolic health groups, CACS did not differ according to obesity status. MUHNO group had a similar level of CACS as MUHO group. Similar results were observed, when the medians for CACS were compared only in subjects with significant coronary artery calcification (CACS>0) (Table S2).

When the proportion of metabolically unhealthy subjects were compared according to degree of coronary artery calcification, the proportion of metabolically unhealthy subjects increased as the CACS increased from 0 to higher than 400 (Figure S2).

### Odds Ratio for Increasing CACS Categories

When multinomial logistic regression analysis with CACS severity categories as the dependent variable and adjusted for age, sex, and smoking status, the odds ratio (OR) for increasing CACS categories increased with OR of MHNO groups being the reference, MHO group being the second lowest, MUHNO group being the second highest and MUHO group being the highest when OR for no calcification status was considered as 1.000 (Table 4). When other metabolic parameters including the components of the metabolic syndrome, which were also used in the categorization of the subjects, were adjusted in the same model, the OR were lowered but still showed similar trends with MUHO group showing the highest OR. Among the parameters, age and smoking showed significantly higher OR for coronary artery calcification compared with other parameters. When the obesity status was defined by WC, multinomial logistic regression analysis revealed similar results compared with the previous analysis with obesity defined by BMI, except that overall OR were lower (Table S3).

**Table 4:** Odds ratio for the increasing CACS categories vs. no calcification in groups divided by metabolic health and obesity with obesity defined by BMI ≥25 kg/m^2^.

CACS categories		1∼10		11∼100		101∼400		>400	
variables	Reference	OR	95% CI	OR	95% CI	OR	95% CI	OR	95% CI
**Model 1**									
Age	+1 year	1.107	1.097∼1.116	1.149	1.140∼1.158	1.205	1.189∼1.221	1.261	1.232∼1.290
Sex	1:men, 2:women	0.262	0.203∼0.338	0.273	0.216∼0.334	0.184	0.118∼0.286	0.149	0.070∼0.314
Smoking	0:no smoking, 1:smoking	1.101	0.967∼1.254	1.295	1.140∼1.470	1.179	0.929∼1.497	1.000	0.629∼1.590
MHNO		1.000	–	1.000	–	1.000	1.000	1.000	–
MHO		1.348	1.129∼1.919	1.250	1.049∼1.489	1.589	1.110∼2.275	1.926	0.911∼4.072
MUHNO		1.513	1.264∼1.811	1.733	1.471∼2.042	2.168	1.569∼2.995	2.926	1.563∼5.480
MUHO		2.229	1.919∼2.589	2.38	2.063∼2.744	3.791	2.856∼5.032	5.221	2.908∼9.374
**Model 2**									
Age	+1 year	1.107	1.097∼1.117	1.150	1.140∼1.159	1.208	1.191∼1.225	1.262	1.232∼1.293
Sex	1:men, 2:women	0.275	0.210∼0.352	0.275	0.217∼0.348	0.195	0.124∼0.307	0.147	0.069∼0.315
SBP	+1 SD	1.139	1.028∼1.261	1.133	1.079∼1.190	1.268	1.122∼1.433	1.237	0.992∼1.542
Calcium	+1 SD	1.012	0.952∼1.075	1.035	0.978∼1.096	1.114	1.002∼1.238	1.036	0.844∼1.272
FBS	+1 SD	1.067	1.003∼1.136	1.083	1.050∼1.118	1.212	1.138∼1.290	1.251	1.104∼1.418
TC	+1 SD	1.283	1.197∼1.377	1.285	1.197∼1.377	1.195	1.039∼1.375	1.331	1.079∼1.641
TG	+1 SD	1.000	1.000∼1.000	1.000	1.000∼1.000	1.100	1.100∼1.100	0.827	0.686∼0.996
Hs-CRP	+1 SD	0.966	0.892∼1.046	1.000	0.952∼1.024	1.005	0.918∼1.099	0.932	0.743∼1.169
Smoking	0:no smoking, 1:smoking	1.077	0.945∼1.227	1.281	1.127∼1.455	1.166	0.916∼1.484	1.016	0.637∼1.620
MHNO		1.000	–	1.000	–	1.000	–	1.000	–
MHO		1.234	1.032∼1.475	1.159	0.971∼1.383	1.433	0.999∼2.055	1.785	0.841∼3.786
MUHNO		1.212	0.996∼1.475	1.447	1.208∼1.734	1.388	0.980∼1.966	2.306	1.165∼4.564
MUHO		1.667	1.397∼1.989	1.896	1.602∼2.245	2.244	1.629∼3.093	3.996	2.053∼7.775

CACS, coronary artery calcium score; OR, odds ratio; CI, confidence interval; MHNO, metabolically healthy non-obese; MHO, metabolically healthy obese; metabolically unhealthy non-obese; metabolically unhealthy obese; SBP, systolic blood pressure; FBS, fasting blood sugar; TC, total cholesterol; TG, triglyceride; Hs-CRP, high-sensitivity C-reactive protein.

## Discussion

In this study performed in a large health-screening cohort, metabolically unhealthy subjects were more prone to coronary artery calcification than metabolically healthy subjects. CACS assessed by MDCT was the highest in metabolically unhealthy obese subjects and the lowest in metabolically healthy non-obese subjects. In post-hoc analysis, there were no significant differences within the same metabolic health groups. There were only significant differences in CACS between the groups divided by metabolic health, suggesting that metabolic health is more associated with coronary artery calcification than obesity. OR for increasing degree of coronary artery calcification were significantly higher in metabolically unhealthy groups with that of MUHO subjects being the highest among the four groups, even after adjusting for other metabolic parameters.

The new concept of “metabolically healthy obesity” stems from the observation that there are subsets of subjects who are obese but without metabolic derangement [19]. These subjects have high BMI, but their metabolic profiles are relatively healthy. There are currently no unique criteria for MHO though several definitions have been proposed [4]. The prevalence of MHO depends on the criteria used [4], and the factors affecting MHO are not yet clearly defined. Among the variables studied, physical activity and waist circumference affected MHO status the most. A recent editorial written by Després et al. [20] suggests a mechanism for better health outcomes despite excess fat in obese patients. The main contributors to metabolic health in this population are the differential distribution of visceral fat and the level of physical activity. Differences in muscle mass, brown fat, subcutaneous fat deposits, and oxidative stress are other explanations for MHO [3]. In our study, a higher proportion of metabolically healthy subjects tended to exercise more compared with metabolically unhealthy counterparts, suggesting the prominent role of physical activity in determining metabolic health in obese patients.

In this study, metabolically unhealthy subjects had significantly worse metabolic parameters compared with metabolically healthy peers; however, LDL-C and total cholesterol levels did not show significant differences between MHO and MUHNO groups. These results suggest that obesity affected total cholesterol and LDL-C levels more than metabolic health in this cohort. High triglycerides and low HDL-C are caused by obesity and insulin resistance [21]. Our study may not capture the true relationship between obesity, metabolic health, and TC and LDL-C levels because the other lipid parameters are already controlled for in this analysis since they are included in the initial categorization. Therefore, after adjustment for HDL-C and TG, total cholesterol and LDL-C levels may be more affected by metabolic health.

Increased attention has been paid to MHO for two reasons. First, different approaches and interventions should be used in subjects with different metabolic health statuses. Second, the protective mechanisms against metabolic derangement in MHO subjects may be applied to metabolically unhealthy subjects. A few studies have reported CVD mortality in metabolically healthy obese subjects [10], [11]. A recent study by Ortega et al. [10] analyzed the association of metabolic health with cardiovascular prognosis and the role of fitness on these analyses in 43,265 adults. They concluded that higher fitness was the most prominent characteristic of MHO subjects, and in 14 years of follow-up, they found that MHO subjects had a lower risk of all-cause mortality and CVD than their metabolically unhealthy obese peers. Additionally, in a 7-year follow-up study of 22,203 subjects, MHO subjects showed no elevated risk for CVD compared with MHNO counterparts, and MUHO subjects showed elevated risk of all-cause mortality compared with MHO counterparts [11]. These results suggest the relative importance of metabolic health over obesity in the development of overt atherosclerosis and mortality.

Subclinical atherosclerosis is important because prevention at this stage would be especially effective [5]. Unfortunately, subclinical atherosclerosis cannot yet be optimally identified. In this study, measurement of coronary artery calcification was used as the detection method for subclinical atherosclerosis. CACS determined by CT is an equivalent measure for coronary atherosclerotic burden in adults and shows good correlation with visceral fat and CVD risk [7]. In this study, CACS was highest in MUHO subjects and lowest in MHNO subjects. Metabolically unhealthy subjects showed higher CACS even when the subject was not obese, suggesting that interventions should be focused on improving metabolic health in this subset.

To our knowledge, this is the first study that has analyzed the association between MHO and CACS in a large population. A study by Khan et al. [12] analyzed the burden of subclinical CVD in 475 metabolically benign and at-risk overweight and obese women. In their study, the risk for coronary artery calcification was significantly increased in metabolically at-risk obese women compared with metabolically benign obese women. The study was limited because its population was relatively small, the health of only female subjects was analyzed, and the definition of metabolic health did not include insulin resistance index. In our study, coronary artery calcification status was studied in more than 25,000 subjects of both genders, subjects on influential medications were strictly excluded, and we adopted all the components that reflect metabolic health, including lipid profiles, hs-CRP, and HOMA-IR as an insulin resistance index. The results of our study suggest that metabolic health is more closely associated with subclinical atherosclerosis assessed by CACS than simply being obese.

In conclusion, in this large, health-screening population, CACS was significantly higher in metabolically unhealthy subjects compared with metabolically healthy subjects regardless of obesity status. In addition, non-obese but metabolically unhealthy subjects showed CACS level similar to metabolically unhealthy obese subjects, suggesting the importance of interventions to improve metabolic health in this subset. Increasing physical activity and reducing visceral fat deposits may be the best way to help this population group. Future research should focus on establishment of a unified definition of metabolic health and validation of these criteria on various study populations.

## Supporting Information

Figure S1 Prevalence of subjects in different CACS categories in different gender
(TIF)

Figure S2 Comparison of proportion of metabolic unhealthy subjects according to degree of coronary artery calcification
(TIF)

Table S1 Comparison of proportion of subjects with CACS higher than 0 among the four groups divided by metabolic health and obesity status
(DOCX)

Table S2 Comparison of medians and interquartile ranges of CACS among the four groups divided by metabolic health and obesity status only in subjects with CACS >0
(DOCX)

Table S3 Odds ratio for the increasing CACS categories vs. no calcification in groups divided by metabolic health and obesity with obesity defined by large waist circumference^*^
(DOCX)
