# Supplementary figures and images for: Metabolic Health Is More Closely Associated with Coronary Artery Calcification than Obesity
Source: PLoS One. 2013 Sep 11;8(9):e74564. doi: 10.1371/journal.pone.0074564 (PMC3770589; doi:10.1371/journal.pone.0074564)

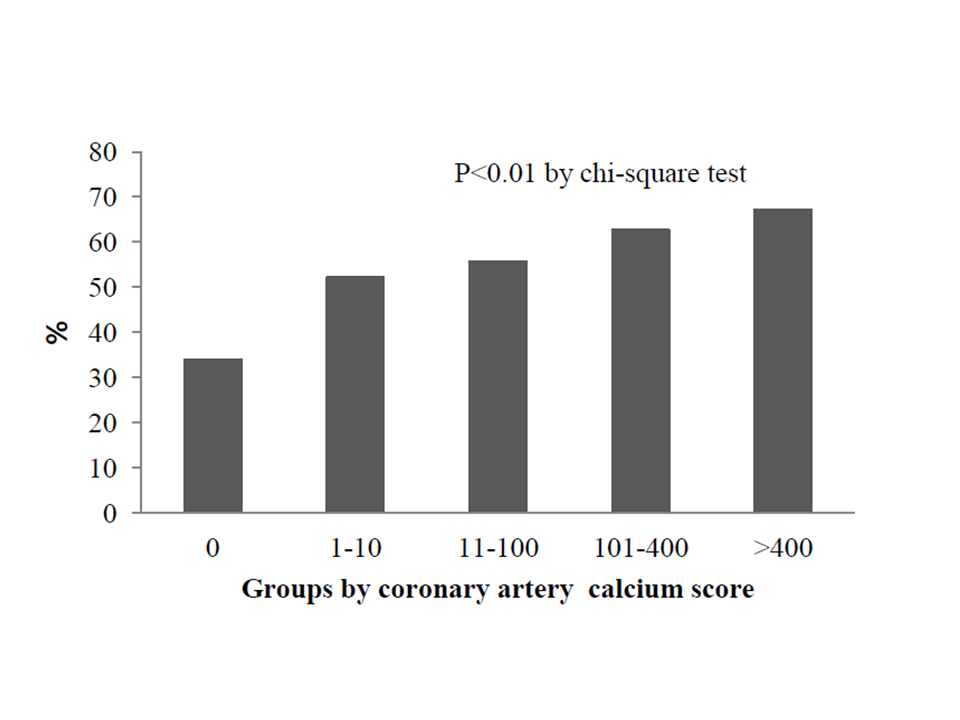

Supplement: Figure S2 — Comparison of proportion of metabolic unhealthy subjects according to degree of coronary artery calcification (TIF) [file pone.0074564.s002.tif]
