# Supplementary material for: Metabolic Health Is More Closely Associated with Coronary Artery Calcification than Obesity
Source: PLoS One. 2013 Sep 11;8(9):e74564. doi: 10.1371/journal.pone.0074564 (PMC3770589; doi:10.1371/journal.pone.0074564)
Supplement: Table S1 — Comparison of proportion of subjects with CACS higher than 0 among the four groups divided by metabolic health and obesity status (DOCX) [file pone.0074564.s003.docx]

**Table S1.** Comparison of proportion of subjects with CACS higher than 0 among the four groups divided by metabolic health and obesity status

| N=24,063 | MHNO | MHO | MUHNO | MUHO | *P* value^*^ |
| --- | --- | --- | --- | --- | --- |
|  | N=10,838 | N=4,389 | N=3,471 | N=5,365 |  |
| Number of subjects with CACS = 0 (%) | 9978 (92.1) | 3898 (88.8) | 2870 (82.7) | 4278 (79.7) | <0.01 |
| Number of subjects CACS > 0 (%) | 860 (7.9) | 491 (11.2) | 600 (17.3) | 1087 (20.3) |  |

^*^P value analyzed by chi-square test

CACS, coronary artery calcium score; MHNO, metabolically healthy non-obese; MHO, metabolically healthy obese; MUHNO, metabolically unhealthy non-obese; MUHO, metabolically unhealthy obese
