# Supplementary material for: Metabolic Health Is More Closely Associated with Coronary Artery Calcification than Obesity
Source: PLoS One. 2013 Sep 11;8(9):e74564. doi: 10.1371/journal.pone.0074564 (PMC3770589; doi:10.1371/journal.pone.0074564)
Supplement: Table S2 — Comparison of medians and interquartile ranges of CACS among the four groups divided by metabolic health and obesity status only in subjects with CACS >0 (DOCX) [file pone.0074564.s004.docx]

**Table S2.** Comparison of medians and interquartile ranges of CACS among the four groups divided by metabolic health and obesity status only in subjects with CACS > 0

| N=3,038 | MHNO (N=860) | MHO (N=491) | MUHNO (N=600) | MUHO (N=1087) | P value^*^ |
| --- | --- | --- | --- | --- | --- |
| Medians of CACS (interquartile range) | 16 (4, 44)^†^ | 14 (4, 51)^†,**^ | 21 (6.25, 67.75)^‡^ | 20 (5, 60)^‡,**^ | <0.01 |

*P value analyzed by Kruskal-Wallis H test

†,‡,** No differences between the groups with same footnotes in post-hoc analyses with Mann-Whitney U test.

CACS, coronary artery calcium score; MHNO, metabolically healthy non-obese; MHO, metabolically healthy obese; MUHNO, metabolically unhealthy non-obese; MUHO, metabolically unhealthy obese
