# Supplementary material for: Metabolic Health Is More Closely Associated with Coronary Artery Calcification than Obesity
Source: PLoS One. 2013 Sep 11;8(9):e74564. doi: 10.1371/journal.pone.0074564 (PMC3770589; doi:10.1371/journal.pone.0074564)
Supplement: Table S3 — Odds ratio for the increasing CACS categories vs. no calcification in groups divided by metabolic health and obesity with obesity defined by large waist circumference* (DOCX) [file pone.0074564.s005.docx]

**Table S3.** Odds ratio for the increasing CACS categories vs. no calcification in groups divided by metabolic health and obesity with obesity defined by large waist circumference^*^

| CACS categories | | 1~10 | | | 11~100 | | 101~400 | | >400 | |
| --- | --- | --- | --- | --- | --- | --- | --- | --- | --- | --- |
| variables | Reference | OR | | 95% CI | OR | 95% CI | OR | 95% CI | OR | 95% CI |
| Model 1 | | | | | | | | | | |
| Age | +1 year | | 1.104 | 1.095~1.114 | 1.147 | 1.138~1.156 | 1.202 | 1.186~1.218 | 1.256 | 1.228~1.285 |
| Sex | 1:men, 2:women | | 0.265 | 0.205~0.342 | 0.280 | 0.221~0.353 | 0.195 | 0.125~0.305 | 0.149 | 0.07~0.317 |
| Smoking | 0:no smoking, 1:smoking | | 1.100 | 0.966~1.253 | 1.289 | 1.135~1.463 | 1.171 | 0.922~1.486 | 0.986 | 0.619~1.571 |
| MHNO |  | |  |  | 1.000 |  | 1.000 |  | 1.000 |  |
| MHO |  | | 1.276 | 1.075~1.516 | 1.279 | 1.082~1.512 | 1.513 | 1.066~2.148 | 1.443 | 0.694~3.000 |
| MUHNO |  | | 1.717 | 1.398~2.110 | 1.829 | 1.505~2.223 | 1.966 | 1.315~2.942 | 3.336 | 1.643~6.775 |
| MUHO |  | | 2.044 | 1.751~2.387 | 2.337 | 2.016~2.709 | 3.69 | 2.738~4.975 | 4.012 | 2.189~7.356 |
| Model 2 | | | | | | | | | | |
| Age | +1 year | | 1.105 | 1.095~1.115 | 1.148 | 1.139~1.158 | 1.205 | 1.188~1.222 | 1.258 | 1.228~1.288 |
| Sex | 1:men, 2:women | | 0.273 | 0.210~0.354 | 0.280 | 0.221~0.355 | 0.204 | 0.129~0.320 | 0.144 | 0.067~0.310 |
| SBP | +1 SD | | 1.147 | 1.066~1.235 | 1.133 | 1.079~1.190 | 1.268 | 1.122~1.433 | 1.284 | 1.030~1.601 |
| Calcium | +1 SD | | 1.007 | 0.947~1.069 | 1.031 | 0.974~1.092 | 1.103 | 0.993~1.225 | 1.023 | 0.834~1.255 |
| FBS | +1 SD | | 1.066 | 1.001~1.135 | 1.083 | 1.050~1.118 | 1.122 | 1.138~1.290 | 1.212 | 1.069~1.374 |
| TC | +1 SD | | 1.284 | 1.197~1.377 | 1.284 | 1.197~1.377 | 1.195 | 1.039~1.375 | 1.331 | 1.079~1.641 |
| TG | +1 SD | | 1.100 | 1.100~1.100 | 1.000 | 1.000~1.000 | 1.100 | 1.100~1.100 | 0.827 | 0.686~0.996 |
| Hs-CRP | +1 SD | | 0.974 | 0.911~1.041 | 1.001 | 0.954~1.051 | 1.007 | 0.920~1.101 | 0.934 | 0.743~1.175 |
| Smoking | 0:no smoking, 1:smoking | | 1.077 | 0.945~1.227 | 1.276 | 1.123~1.450 | 1.159 | 0.910~1.476 | 1.002 | 0.627~1.600 |
| MHNO |  | | 1.000 | - | 1.000 | - | 1.000 | - | 1.000 | - |
| MHO |  | | 1.170 | 0.984~1.391 | 1.188 | 1.004~1.407 | 1.362 | 0.958~1.937 | 1.328 | 0.637~2.768 |
| MUHNO |  | | 1.374 | 1.103~1.711 | 1.526 | 1.239~1.880 | 1.280 | 0.840~1.951 | 2.566 | 1.206~5.463 |
| MUHO |  | | 1.505 | 1.255~1.804 | 1.850 | 1.556~2.201 | 2.163 | 1.548~3.022 | 2.990 | 1.508~5.929 |

CACS, coronary artery calcium score; OR, odds ratio; CI, confidence interval; MHNO, metabolically healthy non-obese; MHO, metabolically healthy obese; MUHNO, metabolically unhealthy non-obese; MUHO, metabolically unhealthy obese; SBP, systolic blood pressure; FBS, fasting blood sugar; TC, total cholesterol; TG, triglyceride; Hs-CRP, high-sensitivity C-reactive protein

* Being obese was defined by different waist circumference cutoffs in different gender for Koreans; men ≥ 90cm, women ≥ 85 cm [18].
